# Scalable risk stratification of undiagnosed heart failure using routine health data and its association with imaging phenotypes and outcomes

**DOI:** 10.1038/s41598-026-60673-w

**Published:** 2026-07-11

**Authors:** Yoko M. Nakao, Ramesh Nadarajah, Farag Shuweihdi, Christopher J. Hayward, Michihiko Goto, Jung-Chi Hsu, Mohammad Haris, Ben Hurdus, Osama Tariq, Temar Habtezghi, Anna Helbitz, Ali Wahab, Lan Mu, Kazuhiro Nakao, Peter Swoboda, Amitava Banerjee, Mark C. Petrie, Clare J. Taylor, Koji Kawakami, Jianhua Wu, Chris P. Gale

**Affiliations:** 1 Leeds Institute of Cardiovascular and Metabolic Medicine, University of Leeds, Leeds, UK; 2 Leeds Institute for Data Analytics, University of Leeds, Leeds, UK; 3 Department of Pharmacoepidemiology, Graduate School of Medicine and Public Health, Kyoto University, Kyoto, Japan; 4 Department of Cardiology, Leeds Teaching Hospital NHS Trust, Leeds, UK; 5 Department of Internal Medicine, University of Iowa Carver College of Medicine, Iowa City, IA USA; 6 Department of Cardiology, National Taiwan University Hospital, Taipei, Taiwan; 7 Wolfson Institute of Population Health, Queen Mary University of London, London, UK; 8 Department of Cardiovascular Medicine, National Cerebral and Cardiovascular Center, Suita, Japan; 9 Department of Biomedical Imaging Science, Leeds Institute of Cardiovascular and Metabolic Medicine, University of Leeds, Leeds, UK; 10 Institute of Health Informatics, University College London, London, UK; 11 School of Cardiovascular and Metabolic Health, University of Glasgow, British Heart Foundation Glasgow Cardiovascular Research Centre, Glasgow, UK; 12 Department of Applied Health Sciences, School of Health Sciences, College of Medicine and Health, University of Birmingham, Birmingham, UK

**Keywords:** Heart failure, Prevention, Prediction, Electronic health records, Screening, Mortality, Cardiology, Diseases, Health care, Medical research, Risk factors

## Abstract

Late diagnosis of Heart failure (HF) is associated with worse outcomes. We aimed to develop a scalable tool to identify those at high risk of undiagnosed HF using routine electronic health records (EHR). We developed and internally validated a logistic regression (FIND-HF) model for incident HF diagnosis within one year in United Kingdom primary care EHRs (CPRD-Aurum, n=3 520 186), with good prediction performance (area under the receiver operating characteristic curve (AUC) 0.79), equal to more complex modelling techniques. We externally validated FIND-HF in United Kingdom (CPRD-GOLD, n=570 850, AUC 0.72), Japan (JMDC, n=6 820 694, AUC 0.73), United States of America (Epic Cosmos, n=7 710 398, AUC 0.78), and Taiwan (NTUH, n=170 518, AUC 0.85). In a cohort who had undergone HF diagnostics an optimised FIND-HF threshold had a positive predictive value of 21.4% and a negative predictive value of 96.9%. Amongst patients with HF who had undergone cardiac magnetic resonance imaging, high FIND-HF risk compared with low FIND-HF risk as reference, was associated with increased risk of a primary composite outcome of heart failure hospitalisation or cardiovascular death and more advanced adverse remodelling including lower left ventricular ejection fraction. FIND-HF is a scalable EHR-based model which has the potential to help rule out undiagnosed HF in low risk cases, whilst high risk cases are associated with more advanced cardiac dysfunction and worse prognosis.

**Supplementary Information:**

The online version contains supplementary material available at https://doi.org/10.1038/s41598-026-60673-w.

## Introduction

Heart failure (HF) affects nearly 60 million people globally,^1^ and following diagnosis prognosis and quality of life is adversely affected.^2,3^ Eight in ten HF cases are diagnosed only after hospitalisation, later in the disease course, reflecting a delayed recognition of the HF syndrome.^4^ Such delays are associated with excess morbidity, mortality, and healthcare expenditure. Early detection of HF in the community and initiation of evidence-based pharmacotherapy may improve quality of life and prognosis.

In 40% of hospital-diagnosed HF cases patients have presented to primary care professionals with symptoms consistent with HF but have not been provided with HF diagnostics.^4^ In older patients with comorbidities, HF diagnosis is delayed, partly because it is considered that symptoms may be related to other conditions (for example obesity, anaemia or chronic obstructive pulmonary disease),^5^ or patients become normalised to their symptoms. Neither the European Society of Cardiology nor American Heart Association / American College of Cardiology / Heart Failure Society of America guidelines recommend screening for HF but recognise earlier detection of left ventricular systolic dysfunction as well as preclinical HF as an evidence gap.^6,7^

Identifying people at risk of undiagnosed HF in the community using routinely available electronic health records (EHRs) could enable more effective targeting of investigations. Our prior systematic review and meta-analysis demonstrated that multivariable prediction models for HF often require variables not routinely available in the community and predict incident HF far in the future, all of which limit their utility for targeting diagnostics in a community setting.^8^

To address this knowledge gap, we conducted a multi-stage study. First we developed and internationally externally validated a prediction model for incident HF within one year that could be applied at scale in community-based EHRs (FIND-HF). We then explored the utility of FIND-HF in a screening context for patients without known HF who underwent HF diagnostics. Finally amongst patients with diagnosed HF we assessed whether high FIND-HF risk, compared with low FIND-HF risk as reference, is associated with worse cardiac structural and functional characteristics and outcomes.

## Results

### Model development and internal validation cohort

There were 3 520 186 individuals in the CPRD Aurum cohort, a cohort of individuals aged ≥40 years with secondary care-linked primary care EHRs in the United Kingdom (UK) without a coded diagnosis of HF, with a mean age of 62.5 years (standard deviation, SD 15.6) and 47.9% women. 37 094 individuals (0.1%) within one year were recorded as having a new diagnosis of HF. These cases of incident HF were older and had a higher prevalence of baseline comorbidities than individuals who did not develop HF (Table 1).

**Table 1:** Baseline characteristics of candidate variables.

	**CPRD Aurum, UK** **(n = 3 520 186)**		**CPRD GOLD, UK** **(n = 570 850)**		**JMDC, Japan** **(n = 6 820 694)**		**Epic Cosmos, US** **(n = 7 710 398)**		**NTUH, Taiwan** **(n = 171 341)**		**FIND-AF cohort, UK** **(n = 255)**	
	**Control**	**HF cases**	**Control**	**HF cases**	**Control**	**HF cases**	**Control**	**HF cases**	**Control**	**HF cases**	**Control**	**HF cases**
**n**	3 483 092	37 094	564 896	5 954	6 772 619	48 075	7 600 925	109 473	170 518	823	224	31
Age	61.5 (15.5)	74.1 (12.9)	69.0 (12.1)	75.3 (11.4)	46.1 (10.3)	53.6 (10.5)	62.1 (12.2)	71.6 (11.8)	65.0 (10.5)	73.5 (11.4)	73.6 (6.3)	78.5 (6.3)
Women	1 666 474 (47.8%)	18 518 (49.9%)	287 080 (50.8%)	2 952 (48.9%)	3 349 358 (49.5%)	20 173 (42.0%)	4 401 483 (47.9%)	55 390 (50.6%)	90 027 (52.8%)	381 (46.3%)	113 (50.4%)	12 (38.7%)
AF	74 987 (2.2%)	4 632 (12.5%)	13 060 (2.3%)	597 (9.9%)	19 730 (0.3%)	2 074 (4.3%)	379 468 (5.0%)	25 753 (23.5%)	7 642 (4.5%)	361 (43.9%)	2 (0.9%)	1 (3.2%)
COPD	84 966 (2.4%)	3 167 (8.5%)	13 975 (2.5%)	507 (8.4%)	5 462 (0.1%)	438 (0.9%)	533 987 (7.0%)	21 742 (19.9%)	5 568 (3.3%)	117 (14.2%)	35 (15.6%)	7 (22.6%)
Depression	300 490 (8.6%)	3 793 (10.2%)	51146 (9.1%)	709 (11.7%)	1 206 (<0.1%)	105 (0.2%)	1 755 131 (23.1%)	31 213 (28.5%)	2 729 (1.6%)	29 (3.5%)	27 (12.1%)	4 (12.9%)
Hypertension	748 324 (21.5%)	14 120 (38.1%)	141 952 (25.1%)	2 145 (35.5%)	779 470 (11.5%)	18 665 (38.8%)	4 755 789 (62.6%)	96 915 (88.5%)	50 226 (29.5%)	577 (70.1%)	188 (83.9%)	28 (90.3%)
Valve diseases	35 066 (1.0%)	1 857 (5.0%)	5 429 (1.0%)	223 (3.7%)	18 313 (0.3%)	1 220 (2.5%)	572 308 (7.5%)	26 156 (23.9%)	3 285 (1.9%)	131 (15.9%)	18 (8.0%)	6 (19.4%)
Vascular diseases	473 426 (13.6%)	15 040 (40.5%)	84 446 (15.0%)	2 232 (36.9%)	238 953 (3.5%)	8 490 (17.7%)	1 582 661 (20.8%)	59 370 (54.2%)	22 667 (13.3%)	444 (53.9%)	40 (17.9%)	11 (35.5%)

Data are mean (standard deviation) or number (%). Vascular diseases include angina, myocardial infarction, stroke, and peripheral vascular diseases.

AF, atrial fibrillation; COPD, chronic obstructive pulmonary disease; CPRD, clinical practice research datalink; FIND-AF, Future Innovations in Novel Detection of Atrial Fibrillation; HF, heart failure.

### Model development and internal validation for prediction of incident HF diagnosis

Across modelling techniques, logistic regression, linear discriminant analysis (LDA) and XGBoost demonstrated similar performance across a combination of discrimination, calibration, and Brier score (Table 2). Calibration intercepts indicated little evidence of systematic over- or underestimation of absolute risk for logistic regression, LDA, and XGBoost, whereas QDA and random forest showed average overestimation. Of the predictive variables, age, vascular disease, and atrial fibrillation (AF) were the most important variables in the model (Supplementary Figure 1). Based on the protocol,^9^ we selected the logistic regression model (FIND-HF) to take forward to external validation.

**Table 2:** Performance for 1-year incident heart failure in CPRD Aurum.

	**Logistic**	**LDA**	**QDA**	**RF**	**XGBoost**
AUROC	0.786 (0.786 – 0.787)	0.788 (0.788 – 0.788)	0.782 (0.781 – 0.782)	0.752 (0.750 – 0.755)	0.794 (0.793 – 0.795)
Brier score	0.010 (0.010 – 0.010)	0.014 (0.014 – 0.014)	0.060 (0.060 – 0.060)	0.018 (0.018 – 0.020)	0.010 (0.010 – 0.010)
Calibration slope	1.029	1.000	0.083	0.248	0.978
Calibration intercept	0	0	-4.15	-2.840	-0.061

Area under received operating characteristic (AUROC) values of <0-60, 60-70, 70-80, and >80 were defined a priori as inadequate, adequate, good, and excellent based on prior publications. The Brier score ranges from 0 to 1, with lower values indicating better predictive accuracy.

AUROC, Area under received operating characteristic; CPRD, Clinical practice research datalink; LDA, Linear discriminant analysis; QDA, Quadratic discriminant analysis; RF, Random forest.

### International external validation for prediction of incident HF diagnosis

We externally validated FIND-HF for prediction of incident HF diagnosis in three external validation cohorts: CPRD-GOLD (a UK-based cohort of secondary care-linked primary care EHRS), JMDC (a claims datasbase in Japan), and Epic Cosmos (an American EHR dataset from hospitals using the Epic system). We included individuals aged ≥40 years without a coded diagnosis of HF. The external validation cohorts included 570 850 individuals in CPRD-GOLD, 6 820 694 individuals in the JMDC cohort, and 7 710 398 in the Epic Cosmos cohort contributing 5 954 (1.0%), 48 075 (0.7%), and 109 473 HF cases (1.4%) within 1 year respectively.

Cases of incident HF were older and had a higher prevalence of baseline comorbidities than individuals who did not develop HF (Table 1). We compared FIND-HF with the Atherosclerosis Risk in Communities (ARIC) HF risk score (age, race, sex, heart rate, systolic blood pressure, blood pressure-lowering medication use, diabetes mellitus, prevalent coronary heart disease, current smoker, former smoker, body mass index)^10^ and Preventive Heart Failure (PCP-HF) model (age, gender, race, current smoking, body mass index, systolic blood pressure [treated of untreated], diabetes mellitus treatment, total cholesterol, high density lipoprotein-cholesterol)^11^.

FIND-HF could be applied to all cases in the CPRD-GOLD, JMDC and Epic Cosmos datasets and demonstrated good discrimination (area under the receiver operating characteristic curve (AUC) in CPRD-GOLD, 0.72; JMDC, 0.73; Epic Cosmos 0.78) (Table 3). However, calibration varied across cohorts. The calibration intercepts indicated average overestimation of absolute risk in CPRD-GOLD and Epic Cosmos, and average underestimation in JMDC. PCP-HF could be applied to 26.7% of participants in JMDC and 1.1% of participants in CPRD-GOLD, whilst ARIC-HF could not be applied in JMDC data (due to absence of heart rate and former smoker) and to 1.7% of participants in CPRD-GOLD. FIND-HF had the best discrimination in CPRD-GOLD and JMDC compared with ARIC-HF and PCP-HF models (Table 3, Supplementary Figure 2). In analyses stratified by sex, FIND-HF model had the best discrimination in both men and women (Supplementary Table 1, Supplementary Figure 2).

**Table 3:** FIND-HF, PCP-HF, and ARIC-HF performance in external validation cohorts.

	**FIND-HF**	**PCP-HF**	**ARIC HF**
**CPRD GOLD, UK**			
AUROC	0.722 (0.715 – 0.728)	0.598 (0.544 – 0.652)	0.599 (0.557 – 0.641)
Brier score	0.010	0.019	0.019
Calibration slope	0.902	0.750	0.596
Calibration intercept	-0.634	0 (fixed)	0 (fixed)
**JMDC, Japan**			
AUROC	0.728 (0.726 – 0.730)	0.702 (0.698 – 0.707)	–
Brier score	0.007	0.008	–
Calibration slope	1.149	0.432	–
Calibration intercept	1.691	–	–
**Epic Cosmos, US**			
AUROC	0.784 (0.782 – 0.785)	–	–
Brier score	0.014	–	–
Calibration slope	0.861	–	–
Calibration intercept	−0.567	–	–

ARIC, Atherosclerosis risk in communities; AUROC, Area under received operating characteristic; FIND-HF, Future Innovations in novel detection of heart failure; PCP-HF, Pooled cohort equations to prevent heart failure.

The Atherosclerosis Risk in Communities (ARIC) HF risk score (age, race, sex, heart rate, systolic blood pressure, blood pressure-lowering medication use, diabetes mellitus, prevalent coronary heart disease, current smoker, former smoker, body mass index)^10^ and Preventive Heart Failure (PCP-HF) model (age, gender, race, current smoking, body mass index, systolic blood pressure [treated of untreated], diabetes mellitus treatment, total cholesterol, high density lipoprotein-cholesterol)^11^ are models previously developed and validated for prediction of incident HF.^8^

### International external validation for prediction of incident HF hospitalisation

We specifically tested FIND-HF for prediction of incident HF hospitalisation in patients without a known diagnosis of HF using the National Taiwan University Hospital (NTUH) EHR dataset. In the Taiwan cohort, of 170 518 patients aged ≥40 years without coded HF diagnosis there were 823 admissions (1.6%) to hospital with HF within one year. Patients with incident HF hospitalisation were older and a higher prevalence of comorbidities than patients who did not have an incident HF hospitalisation (Table 1). FIND-HF demonstrated excellent prediction performance for HF hospitalisation within one year for patients without prevalent diagnosis of HF (AUC 0.85, 95% CI 0.84-0.86) (Supplementary Table 2). Prediction performance was excellent in both men and women (Supplementary Table 3).

### Clinical utility modelling for FIND-HF to guide HF screening

The FIND-AF cohort is a sub-study where patients at high risk of undiagnosed AF without known HF underwent HF assessment with symptom assessment and diagnostic tests. Of 224 participants without known HF, diagnostic testing identified 31 new cases of HF, including four cases of heart failure with reduced ejection fraction (HFrEF), four cases of heart failure with mid-range ejection fraction (HFmrEF), and 23 cases of heart failure with preserved ejection fraction (HFpEF). Patients diagnosed with HF had a higher burden of cardiovascular comorbidities and were more commonly men compared with participants who were not diagnosed with HF (Table 1). The prediction performance of FIND-HF for the composite of HFrEF, HFmrEF, HFpEF and asymptomatic left ventricular systolic dysfunction was AUC 0.77 (95% CI 0.69-0.85). At an optimised threshold of 0.0149 (based on the highest F1 score of 0.344) FIND-HF had a positive predictive value of 0.214 and a negative predictive value 0.969 (Supplementary Table 4).

### Clinical outcomes and cardiac structural and functional characteristics of HF cases with high FIND-HF score compared with low FIND-HF score

The MATCH (MyocArdial Tissue CHaracteristics in patients with HF according to glycaemic status) 1 and 2 registries recruited patients with a new diagnosis of HF who were referred for cardiac magnetic resonance (CMR) imaging in SeeFig. 1 routine care. Amongst 553 patients (mean age 64.4 years (SD 10.3), 192 (34.7%) women), 263 had high FIND-HF risk scores compared with 290 with low FIND-HF risk score scores, using the optimised threshold of 0.0149 from the clinical utility modelling analysis (Supplementary Table 5). Patients with high FIND-HF risk were older and had a higher prevalence of vascular disease and valvular heart disease (Supplementary Table 5).

**Fig. 1:**
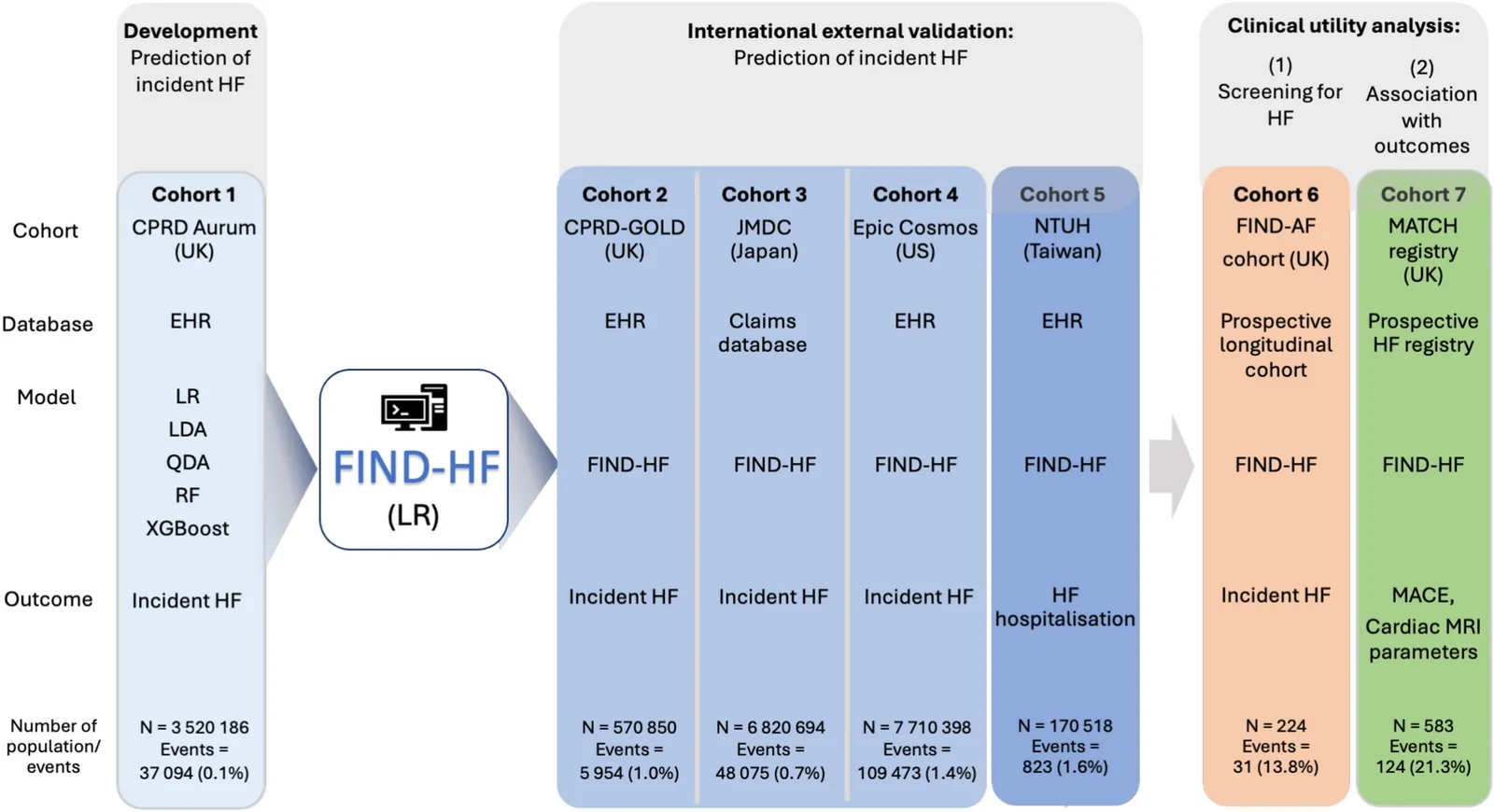
Flow diagram of cohorts in study CPRD, Clinical Practice Research Datalink; EHR, electronic health record; FIND-AF, Future Innovations in Novel Detection of Atrial Fibrillation; FIND-HF, Future Innovations in Novel Detection of Heart Failure; HF, heart failure; LDA, Linear Discriminant Analysis; LR, logistic regression; MACE, major adverse cardiovascular events; MATCH, MyocArdial Tissue CHaracteristics in patients with HF according to glycaemic status; MRI, magnetic resonance imaging; NTUH, National Taiwan University Hospital; QDA, Quadratic Discriminant Analysis; RF, Random Forest. This multi-stage population-based study included: (i) development and internal validation of the prediction model for incident HF diagnosis in CPRD-AURUM (UK), (ii) multi-national external validation for incident HF diagnosis in CPRD-GOLD (UK), JMDC (Japan) and Epic Cosmos (USA), (iii) international external validation for HF hospitalization in NTUH (Taiwan), (iv) modelling of clinical utility for targeting HF diagnostics in a prospective cohort who had undergone HF diagnostics (FIND-AF, UK), and (v) estimation of outcomes, and structural and functional cardiac MRI characteristics in patients with HF in the MATCH registry (UK) stratified by FIND-HF score.

#### Outcomes

**Fig. 2:**
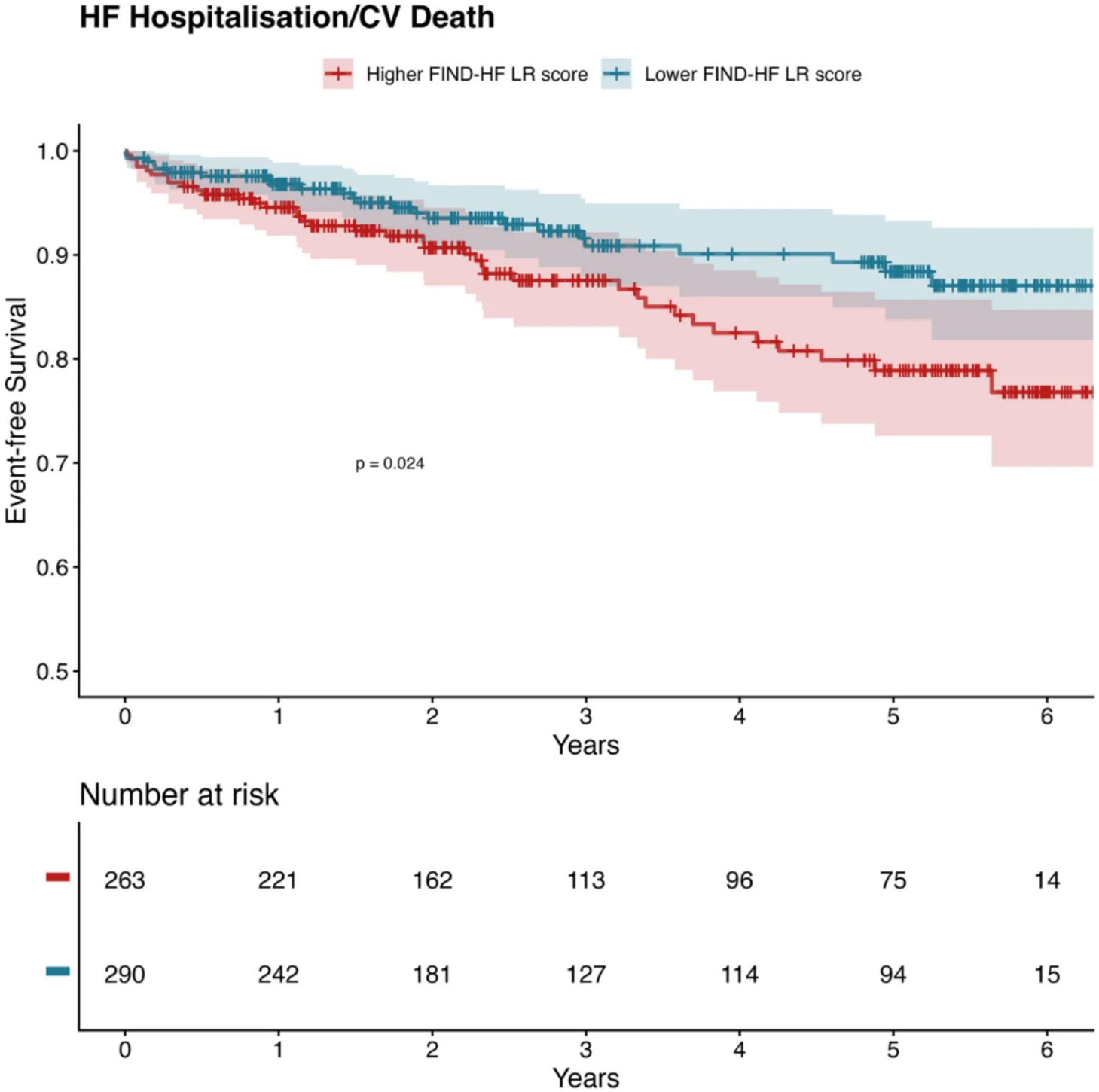
Kaplan-Meier plots of the primary composite outcome of heart failure hospitalisation or cardiovascular death for patients with HF in the MATCH registry stratified by FIND-HF score. HF, heart failure; CV, cardiovascular; LR, logistic regression; MACE, major adverse cardiovascular event (composite of myocardial infarction, stroke, heart failure hospitalisation, and death); MATCH, MyocArdial Tissue CHaracteristics in patients with HF according to glycaemic status. High FIND-HF risk refers to patients with HF with a FIND-HF score of ≥0.0149 based on the optimised threshold from the clinical utility modelling analysis. Kaplan–Meier curves were compared using the log-rank test, and crude hazard ratios (HRs) with 95% CI were estimated using univariable Cox proportional hazards regression. Patients with HF and high FIND-HF risk more frequently experience the primary composite outcome than patients with HF with low FIND-HF risk.

The primary outcome was time to a composite of heart failure hospitalisation or cardiovascular death which occurred in 38 (14.5%) individuals in the high FIND-HF risk group, and in 24 (8.3%) individuals in the low FIND-HF risk group (HR 1.78, 95% CI 1.07-2.98, p = 0.026) over median 2.6 (interquartile range 1.4-5.1) years follow-up (Fig. 2, Supplementary Table 6-7).

Of the components of the primary outcome HF hospitalisation occurred in 32 (12.2%) patients in the high FIND-HF risk group and 20 (6.9%) patients in the low FIND-HF risk group (HR 1.79, 95% CI 1.03-3.13, p = 0.041) (Supplementary Figure 2). Sensitivity analysis censoring follow-up at 5 years showed consistent results (Supplementary Figure 3, Supplementary Table 8-9).

#### Cardiac MRI characteristics

Patients with high FIND-HF risk had a greater prevalence of ischaemic late gadolinium enhancement suggestive of prior myocardial infarction (30.4% vs 12.4%, p = 0.004) and inducible ischaemia (12.9% vs 3.1%, p = 0.024), larger indexed left atrial volume (51.8 vs 40.2 ml, p<0.001), and lower left ventricular ejection fraction (36.8% vs 40.6%, p = 0.038) (Supplementary Table 10).

A higher FIND-HF score was associated with lower left ventricular ejection fraction (regression coefficient β = -3.71, 95% CI -5.86 to -1.56, p<0.001), higher cardiac MRI-derived pulmonary capillary wedge pressure (0.71, 95% CI 0.25 to 1.18, p=0.003), higher indexed left atrial volume (11.51, 95% CI 8.07 to 14.95, p<0.001), lower right ventricular ejection fraction (-4.57, 95% CI -6.57 to -2.57, p<0.001), as well as higher extracellular volume (1.21, 95% CI 0.37 to 2.06, p=0.005) and higher native T1 (10.63, 95% CI 1.51 to 19.74, p=0.022) (Supplementary Table 11 and Supplementary Figure 4).

## Discussion

We developed and validated in almost 19 million patients from internationally diverse cohorts a multivariable model to identify individuals at higher risk of undiagnosed HF and assessed its clinical utility and association with clinical outcomes and functional and structural pathology. FIND-HF was found to be scalable in routinely collected EHRs, to produce good risk stratification for HF and HF hospitalisation within the next one year for patients without known HF, and to be associated with a worse cardiac structural and functional phenotype at increased risk of a primary composite outcome of heart failure hospitalisation or cardiovascular death.

Our previous systematic review and meta-analysis summarised a plethora of models developed and/or validated to predict incident HF, and the best-performing were the ARIC-HF and PCP-HF models, both of which predicted incident HF in 5- to 10-years.^10,12^ This study is in sharp contrast to previous prediction modelling studies in HF in having a shorter prediction horizon of one year. Thus, the aim was to identify individuals with likely undiagnosed but manifest HF. In the clinical utility modelling study, recruiting patients at high FIND-HF risk without further enrichment factors such as symptoms or elevated natriuretic peptides detected a new HF diagnosis in 1 in 5 patients. Of patients with HF symptoms presenting in primary care, only 39% receive natriuretic peptide testing,^13^ and the median time from symptom to diagnostics is over nine months, with the time between symptom presentation to HF diagnosis being over two and a half years.^13^ With its high negative predictive value FIND-HF could be used to rule out HF testing in patients stratified as low risk, streamlining clinical pathways. Alternatively, it could guide as an enrichment criteria for community-based screening for HF, in a health system aiming to address the current large burden of undiagnosed HF in community-dwelling individuals. However, before one considers implementing FIND-HF prospective randomised assessment is required to assess its impact on rates of HF diagnosis and clinical outcomes.

Notably, the majority of new HF diagnoses in the clinical utility stage of the study were of HFpEF. HFpEF differs from HFrEF and HFmrEF in that patients are older, more often women, and have a higher prevalence of non-cardiovascular comorbidities^14^. HFpEF is estimated to become the most common type of HF,^15^ and whilst the rate of hospitalisation for HFrEF is in decline, for HFpEF it is increasing.^16^ Following recent randomised clinical trials, the evidence base for treatment of HFpEF has expanded to include sodium-glucose cotransporter 2 inhibitors and finerenone, as well as glucagon-like peptide-1 receptor agonists for patients with HFpEF and obesity.^17–19^ It is possible that HFpEF represents the majority of undiagnosed HF given that it will often present in patients with competing reasons to be symptomatic. As there are now opportunities to improve the care and outcomes of patients with HFpEF if diagnosis is achieved earlier, risk-guided case finding could be particularly helpful to accelerate earlier diagnosis and treatment of HFpEF.

Alternative approaches have been studied for identifying undiagnosed HF risk. A study within the Mayo Clinic network showed that higher risk identified by artificial intelligence analysis of ECGs was associated with increased diagnosis of left ventricular systolic dysfunction, but the utility of this approach outside this specific hospital network is unknown.^20^ The STOP-HF randomised clinic trial showed that BNP-based screening and collaborative care reduced the combined rates of LV systolic dysfunction, diastolic dysfunction, and HF,^21^ but implementing this at scale would require point of care testing - adding expense and inconvenience. Large language models within routine EHRs may provide accurate risk stratification for HF screening, though there are complexities for implementation including resources, hallucinations, as well as integration and privacy challenges.^22^ By contrast the variables incorporated within FIND-HF are recognised to be implicated in HF genesis, which should engender trust of clinicians in risk estimation provided, and regression models can be implemented through currently available EHR architectures.

Our work is best understood in the context of its limitations. First, the CPRD, JMDC, Epic Cosmos, and NTUH databases are routinely collected, retrospective data. Underestimation of HF incidence is possible since there will have been individuals with unrecorded asymptomatic HF. ICD code usage practices that we used for comorbidities and outcome evaluation also vary across hospitals and nations, which may affect the consistency of diagnoses. Second, predictor variables that may have improved prediction performance were excluded due to missingness in routine data as we purposely developed FIND-HF to be scalable. Third, although existing ARIC-HF and PCP-HF models could, in theory, be applied to Epic Cosmos for comparative validation, we chose not to do so due to resource limitations. We plan to address comparative performance in various populations in future work. Fourth, clinical utility analysis was conducted in the FIND-AF cohort who had an absence of atrial fibrillation, which is an important predictor for HF, and were a population that had been selected as high risk of undiagnosed AF, and therefore do not reflect an all-comer population. The positive predictive value of FIND-HF may be different in an all-comer population and is the subject of prospective study (BHF, PG/25/12417). Fifth, though high FIND-HF risk was associated with adverse outcomes in patients with HF, the magnitude of risk may differ in screen-detected HF, and the MATCH registry is not reflective of all HF patients as these patients had been referred for CMR during routine care. Sixth, our model deliberately uses routinely available demographics and diagnosis-code–derived indicators, and we observed no missing predictor data in the present analytical cohorts. However, implementation in other health systems may encounter occasional data incompleteness due to local capture, coding, or extraction workflows. The real-world impact of such operational issues (e.g., missing demographics, interrupted coding feeds, or delayed code availability) and the feasibility of site-specific governance rules should be assessed in prospective implementation studies. Furthermore, differences in healthcare policy, referral pathways, coding practices, and access to diagnostics across countries may influence calibration and implementation of FIND-HF and requires prospective assessment in individual healthcare systems.

In conclusion, FIND-HF shows good-to-excellent prediction performance for incident HF and HF hospitalisation across international routine EHR cohorts within the next year amongst patients without known HF, and can be applied at scale. Prospective evaluation is now required to assess whether use of FIND-HF can improve community-based diagnosis of HF and reduce adverse outcomes.

## Methods

### Study design

This multi-stage population-based study comprised several sequential analytical stages, including: (i) development and internal validation of the prediction model for undiagnosed HF, (ii) multi-national external validation, (iii) external validation for HF hospitalisation, (iv) modelling of clinical utility for targeting HF diagnostics in a prospective cohort, and (v) estimation of outcomes, and structural and functional cardiac MRI characteristics in patients with high FIND-HF score and HF diagnosed (Fig. 1). This study evolved from the previously published methods due to availability of additional datasets.^9^ We followed the Transparent Reporting of a Multivariable Prediction Model for Individual Prognosis and Diagnosis + Artificial Intelligence (TRIPOD+AI) statement.^23^

### Data sources

We developed the model in CPRD Aurum (UK primary care EHR linked to HES Admitted Patient Care and ONS Death Registration) and conducted international external validation in CPRD GOLD (UK), JMDC Claims Database (Japan), and Epic Cosmos (USA), with additional evaluation in NTUH-iMD (Taiwan). Cohort entry windows were January 1998–February 2022 for CPRD datasets, January 2005–October 2023 for JMDC, and January 2021–December 2024 for Epic Cosmos; NTUH-iMD comprised routinely collected hospital EHRs (dataset details in the Supplementary Methods). To examine phenotypes and outcomes associated with FIND-HF risk, we analysed two independent cohorts. First, the FIND-AF longitudinal cohort (UK) recruited community participants identified as at risk of AF, who then underwent cross-sectional phenotyping including symptom assessment, natriuretic peptide testing and transthoracic echocardiography (October 2023–November 2024). Second, the MATCH 1 and MATCH 2 registries enrolled adults (≥40 years) with clinically diagnosed HF referred for cardiac MRI between 2018 and 2024; cardiac MRI was performed using a standardised 3T protocol, and outcomes (primary: composite MACE of HF hospitalisation, all-cause mortality, stroke and myocardial infarction) were ascertained from participant-level medical records (full inclusion/exclusion criteria and imaging protocol in the Supplementary Methods).

### Ethics approval and consent to participate

All methods were carried out in accordance with relevant guidelines and regulations. The use of UK primary care data (CPRD Aurum and CPRD GOLD) was approved by the Independent Scientific Advisory Committee (ISAC) (ref. 21_000324). Use of the JMDC Claims Database was approved by the Kyoto University Institutional Review Board (No. R4368). Use of NTUH-iMD was approved by the National Taiwan University Hospital IRB (202410100RINA). The FIND-AF longitudinal cohort received approval from the North West – Greater Manchester South Research Ethics Committee (23/NW/0180). The MATCH 1/2 registries were approved by the National Research Ethics Committee (17/YH/0300 and 20/NW/0326). Written informed consent was obtained from all participants in the FIND-AF cohort and the MATCH registries. For analyses of anonymised or de-identified routinely collected records (CPRD Aurum/GOLD, JMDC, NTUH-iMD, Epic Cosmos), the requirement for informed consent was waived. Epic Cosmos contains only de-identified data in accordance with Health Insurance Portability and Accountability Act and is used under strict data-use policies within approved environments.

### Model development and internal validation

We included all men and women registered at primary practices who were ≥40 years of age at entry with no history of HF and had at least one year of follow-up, between January 1998 and February 2022 in CPRD Aurum (more details of data source in appendix).^24^ Patients were followed until a diagnosis of HF, death, withdrawal from the database, or one year, whichever came first. We defined incident HF as the first record of HF in primary care (using SNOMED CT codes) or hospital admission records (using the International Classification of Diseases, tenth revision [ICD-10]) from any diagnostic position using a comprehensive set of diagnostic codes.^25,26^

Candidate predictor variables were selected a priori from a systematic review of variables included in previous HF risk multivariable prediction models.^8^ To create a model that could be implemented at scale in national primary care EHRs (and thus be robust to missing data), we restricted candidate variables to age, sex and comorbidities, which were coded as binary parameter indicating the presence or absence of recorded diagnoses and excluded the need for clinical observations and laboratory investigations. We chose eight variables: age, sex, atrial fibrillation, chronic obstructive pulmonary disease, hypertension, vascular diseases (angina, myocardial infarction, stroke, and peripheral vascular disease), valvular heart disease, and depression to be used in the models. We assessed additional candidate comorbidities during model development; however, diabetes mellitus provided minimal incremental predictive value in our data and did not materially improve overall model performance. We also excluded obesity because it is often under-recorded in routine EHR/claims data unless it prompts specific clinical management, so the absence of an obesity code does not reliably reflect the absence of obesity and would risk substantial misclassification. Concordantly, our entire analytical cohort had no missing data for any of the predictor variables, and the model could be applied to all cases in each of the EHRs.

We evaluated five types of models: logistic regression, random forest, linear discriminant analysis, quadratic discriminant analysis, and XGBoost. For the random forest model, hyperparameters were explicitly tuned to prevent overfitting by setting the number of variables at each split to 3 and the minimum leaf size to 5, with 1,000 trees grown to ensure stability. Additionally, we employed stratified sampling and prevalence-adjusted voting cutoffs to address the severe class imbalance. Internal validation of the models was performed using bootstrapping with 200 samples to evaluate their performance. The area under the received operating characteristic curve (AUROC) was used to evaluate predictive ability (concordance index) with 95% confidence intervals (CIs) calculated using the DeLong method. AUROC values of <0-60, 60-69, 70-80, and >80 were defined a priori as inadequate, adequate, good, and excellent based on prior publications.^27^ Calibration was assessed by the calibration slope; notably, for the random forest model, a deviation from unity was anticipated as a trade-off for prioritizing the discrimination of rare events. We calculated the Brier score, a measure of both discrimination and calibration. Sample size calculations are discussed in the appendix p.6. We took the best performing model forward for external validation, prioritising lower complexity and more implementable methods in the event of equal performance.^9^

### International external validation for prediction of incident HF diagnosis

We included all men and women registered who were ≥40 years of age with no history of HF and had at least one year of follow-up from Clinical Practice Research Datalink-GOLD (CPRD-GOLD, UK), which comprises a population entirely distinct from that of CPRD-Aurum, between January 1998 and February 2022,^28^ from JMDC Claims Database (JMDC, Japan) between January 2005 and October 2023^29^, and Epic Cosmos (USA) between January 2021 and December 2024.^30^ The details of the included datasets are described in the appendix pp.3-4. Incident HF was identified by ICD-10 codes (CPRD-GOLD, JMDC, Epic Cosmos) and Read codes (CPRD-GOLD).^9^

Performance was assessed with AUROC, calibration slope, and Brier score and compared with the Atherosclerosis Risk in Communities (ARIC) HF risk score (age, race, sex, heart rate, systolic blood pressure, blood pressure-lowering medication use, diabetes mellitus, prevalent coronary heart disease, current smoker, former smoker, body mass index)^10^ and Preventive Heart Failure (PCP-HF) model (age, gender, race, current smoking, body mass index, systolic blood pressure [treated of untreated], diabetes mellitus treatment, total cholesterol, high density lipoprotein-cholesterol)^11^, because these were the best-performing models identified in our systematic review and meta-analysis for prediction of incident HF.^8^ However both of these models predict development of HF at longer prediction horizons (5 or 10 years). For ARIC-HF and PCP-HF, we calculated the proportion of patients for whom the models could be applied without imputation of missing data. We calculated prediction performance metrics in the subpopulations without requirement for imputation. We investigated the performance of these two models and FIND-HF according to sex.

### International external validation for prediction of incident HF hospitalisation

We included all men and women who were ≥40 years of age at entry with no history of HF and had at least one year of follow-up after attendance at the National Taiwan University Hospital (NTUH, details of dataset in the appendix p.3).^31^ We identified incident HF hospitalisation using ICD-10 codes and including cases from the primary diagnostic position. FIND-HF was applied to the NTUH database and performance assessed with AUROC, calibration slope, and Brier score. We conducted subgroup analysis by sex.

### Clinical utility modelling for FIND-HF to guide HF screening

We included all participants without a history of HF who underwent symptom assessment, natriuretic peptide testing and transthoracic echocardiography as part of the FIND-AF longitudinal cohort study (details in appendix p.4).^32^ New diagnoses of HF with reduced ejection fraction (HFrEF), HF with mid-range ejection fraction (HFmrEF), HF with preserved ejection fraction (HFpEF) and asymptomatic left ventricular systolic dysfunction were made in accordance with European Society of Cardiology guidelines.^6^ FIND-HF was evaluated with AUROC, sensitivity, specificity, positive predictive value and negative predictive value for HF diagnosis.^26^ To ensure clinical reproducibility and utility, we identified an optimum probability threshold to define a "high-risk" group. This threshold was determined by calculating the score that provided the optimum yield for HF screening within the FIND-AF longitudinal cohort and the F1 score. The probability threshold was chosen by maximizing the F1 score, defined as the harmonic mean of precision and recall, which identifies the cutoff that optimally balances precision and recall for classifying positive cases. Based on this analysis, a FIND-HF logistic regression score of 0.0149 was established as the optimal cutoff for identifying individuals at high risk.

### Clinical outcomes and cardiac structural and functional characteristics of HF cases with high FIND-HF score compared with low FIND-HF score

We included all participants aged ≥40 years, diagnosed with HF in the previous year, referred for cardiac MRI assessment, and recruited into the MATCH (MyocArdial Tissue CHaracteristics in patients with HF according to glycaemic status) 1 and MATCH 2 registries between 2018 and 2024.^33^ Details of the MATCH registries are provided in appendix p.4. The primary outcome was HF hospitalisation or cardiovascular death as derived from participant-level medical records. Secondary outcomes were the components of the primary outcome and the association of cardiac MRI variables with FIND-HF score. This analysis was intended as an exploratory assessment of whether a higher FIND-HF score was associated with subsequent clinical outcomes and adverse cardiac structural and functional characteristics among patients with established HF, rather than as an assessment of prognostic model performance in the MATCH cohort.

Patients were followed from the date of cardiac MRI until the occurrence of the outcome of interest, death or last chart review, whichever came first. Kaplan–Meier plots were constructed to visualise time-to-event outcomes with 10 or more events, stratified by high and low FIND-HF risk scores defined by the optimum threshold identified in the clinical utility modelling stage. Kaplan–Meier curves were compared using the log-rank test, and crude hazard ratios (HRs) with 95% CI were estimated using univariable Cox proportional hazards regression to describe unadjusted associations in this exploratory analysis and avoid potential overadjustment from covariates already incorporated within the FIND-HF score. For analyses of cardiovascular death, non-cardiovascular death was treated as a censoring event using a cause-specific hazard model. Additional sensitivity analyses were performed with follow-up censored at 5 years.

To evaluate whether higher FIND-HF scores were associated with more structural and functional abnormalities in the MATCH registry, we dichotomised patients into high and lower FIND-HF risk using the optimum thresholds. Cardiac MRI characteristics were compared descriptively between groups using chi-square test or Fisher’s exact test for categorical variables and t-test for continuous variables, as appropriate. Univariable linear regression analyses were then performed separately for each continuous cardiac MRI variable to examine unadjusted associations between FIND-HF risk group and cardiac MRI measures.

We used STATA, version 17.0 and R 4.3.1 to perform statistical analysis with statistical significance set at P< 0.05.

## Author’s contributors

YMN, RN, KN, JW and CPG conceived the idea of the study. YMN, FS, CJH, MG, JH and LM undertook data extraction, data cleaning and statistical analyses. YMN, RN, JW and CPG interpreted the findings and YMN drafted the manuscript. RN, FS, CJH, MG, JH, MH, BH, OT, TH, AH, AW, LM, KN, PS, AB, MP, CJT, KK, JW and CPG critically reviewed the manuscript and YMN and RN revised the manuscript for final submission. YMN, RN, FS, CJH, KN, JW and CPG were eligible to access the raw CPRD data for model development and validation. YMN and KK were eligible to access JMDC data, MG and YMN were eligible to access Epic Cosmos data, RN, FS and JH were eligible to access Taiwan data, RN, FS, and JW were eligible to access the FIND-AF data, and RN, FS, CJH, LM, and JW were eligible to access the MARCH registry data. All authors accept the final responsibility to submit for publication.

## Supplementary Information

Supplementary Information.

## Data Availability

The authors do not have permission to share data. Data may be obtained from a third party and are not publicly available. The model can be shared with researchers who agree to use it only for research purposes with a data sharing agreement.

## References

[1] Yan, T. et al. Burden, trends, and inequalities of heart failure globally, 1990 to 2019: a secondary analysis based on the Global Burden of Disease 2019 Study. *J. Am. Heart Assoc.* **12**, e027852. 10.1161/JAHA.122.027852 (2023). PMC10111559 36892088

[2] Taylor, C. J. et al. Trends in survival after a diagnosis of heart failure in the United Kingdom 2000–2017: population based cohort study. *BMJ* **364**, l223. 10.1136/bmj.l223 (2019). PMC6372921 30760447

[3] Conrad, N. et al. Temporal trends and patterns in mortality after incident heart failure: a longitudinal analysis of 86 000 individuals. *JAMA Cardiol.* **4**, 1102–1111 (2019). 10.1001/jamacardio.2019.3593 PMC6724155 31479100

[4] Bottle, A. et al. Routes to diagnosis of heart failure: observational study using linked data in England. *Heart* **104**, 600–605 (2018). 10.1136/heartjnl-2017-312183 28982720

[5] Fry, M. et al. The implications of living with heart failure; the impact on everyday life, family support, co-morbidities and access to healthcare: a secondary qualitative analysis. *BMC Fam. Pract.* **17**, 139. 10.1186/s12875-016-0537-5 (2016). PMC5037641 27670294

[6] Members:, A. T. F. *et al.* 2021 ESC Guidelines for the diagnosis and treatment of acute and chronic heart failure: Developed by the Task Force for the diagnosis and treatment of acute and chronic heart failure of the European Society of Cardiology (ESC). With the special contribution of the Heart Failure Association (HFA) of the ESC. *European journal of heart failure* **24** 4-131 (2022). 10.1002/ejhf.2333 35083827

[7] Heidenreich, P. A. et al. 2022 AHA/ACC/HFSA guideline for the management of heart failure: a report of the american college of cardiology/american heart association joint committee on clinical practice guidelines. *Circulation* **145**, e895–e1032. 10.1161/cir.0000000000001063 (2022). 35363499

[8] Nadarajah, R. et al. Prediction models for heart failure in the community: a systematic review and meta-analysis. *Eur. J. Heart Fail.* **25**, 1724–1738 (2023). 10.1002/ejhf.2970 37403669

[9] Nakao, Y. M. et al. Predicting incident heart failure from population-based nationwide electronic health records: protocol for a model development and validation study. *BMJ Open* **14**, e073455. 10.1136/bmjopen-2023-073455 (2024). PMC10806764 38253453

[10] Agarwal, S. K. et al. Prediction of incident heart failure in general practice: the atherosclerosis risk in communities (ARIC) study. *Circ. Heart Fail.* **5**, 422–429. 10.1161/circheartfailure.111.964841 (2012). PMC3412686 22589298

[11] Bavishi, A. et al. Predictive accuracy of heart failure-specific risk equations in an electronic health record-based cohort. *Circ. Heart Fail.* **13**, e007462. 10.1161/circheartfailure.120.007462 (2020). PMC7674211 33092406

[12] Khan, S. S. et al. 10-year risk equations for incident heart failure in the general population. *J. Am. Coll. Cardiol.* **73**, 2388–2397. 10.1016/j.jacc.2019.02.057 (2019). PMC6527121 31097157

[13] Hayhoe, B. et al. Adherence to guidelines in management of symptoms suggestive of heart failure in primary care. *Heart* **105**, 678–685 (2019). 10.1136/heartjnl-2018-313971 30514731

[14] Borlaug, B. A. Evaluation and management of heart failure with preserved ejection fraction. *Nat. Rev. Cardiol.* **17**, 559–573 (2020). 10.1038/s41569-020-0363-2 32231333

[15] Banerjee, P. Vol. 204 198-199 (Elsevier, 2016).

[16] Oktay, A. A., Rich, J. D. & Shah, S. J. The emerging epidemic of heart failure with preserved ejection fraction. *Curr. Heart Fail. Rep.* **10**, 401–410 (2013). 10.1007/s11897-013-0155-7 PMC3870014 24078336

[17] Solomon, S. D. et al. Dapagliflozin in heart failure with mildly reduced or preserved ejection fraction. *N. Engl. J. Med.* **387**, 1089–1098. 10.1056/NEJMoa2206286 (2022). 36027570

[18] Anker, S. D. et al. Empagliflozin in heart failure with a preserved ejection fraction. *N. Engl. J. Med.* **385**, 1451–1461. 10.1056/NEJMoa2107038 (2021). 34449189

[19] Kosiborod, M. N. et al. Semaglutide in patients with heart failure with preserved ejection fraction and obesity. *N. Engl. J. Med.* **389**, 1069–1084. 10.1056/NEJMoa2306963 (2023). 37622681

[20] Attia, Z. I. et al. Screening for cardiac contractile dysfunction using an artificial intelligence-enabled electrocardiogram. *Nat. Med.* **25**, 70–74. 10.1038/s41591-018-0240-2 (2019). 30617318

[21] Ledwidge, M. et al. Natriuretic peptide-based screening and collaborative care for heart failure: The STOP-HF randomized trial. *JAMA* **310**, 66–74. 10.1001/jama.2013.7588 (2013). 23821090

[22] Dennstädt, F., Hastings, J., Putora, P. M., Schmerder, M. & Cihoric, N. Implementing large language models in healthcare while balancing control, collaboration, costs and security. *NPJ Digit. Med.* **8**, 143 (2025). 10.1038/s41746-025-01476-7 PMC11885444 40050366

[23] Collins, G. S. *et al.* TRIPOD+ AI statement: updated guidance for reporting clinical prediction models that use regression or machine learning methods. *bmj* **385** (2024). 10.1136/bmj-2023-078378 PMC11019967 38626948

[24] Wolf, A. et al. Data resource profile: Clinical Practice Research Datalink (CPRD) Aurum. *Int. J. Epidemiol.* **48**, 1740–1740g. 10.1093/ije/dyz034 (2019). PMC6929522 30859197

[25] Conrad, N. et al. Temporal trends and patterns in heart failure incidence: a population-based study of 4 million individuals. *Lancet* **391**, 572–580. 10.1016/s0140-6736(17)32520-5 (2018). PMC5814791 29174292

[26] Nakao, Y. M. et al. Prognosis, characteristics, and provision of care for patients with the unspecified heart failure electronic health record phenotype: a population-based linked cohort study of 95262 individuals. *EClinicalMedicine* **63**, 102164. 10.1016/j.eclinm.2023.102164 (2023). PMC10474358 37662516

[27] Lloyd-Jones, D. M. Cardiovascular risk prediction: basic concepts, current status, and future directions. *Circulation* **121**, 1768–1777 (2010). 10.1161/CIRCULATIONAHA.109.849166 20404268

[28] Herrett, E. et al. Data resource profile: Clinical Practice Research Datalink (CPRD). *Int. J. Epidemiol.* **44**, 827–836. 10.1093/ije/dyv098 (2015). PMC4521131 26050254

[29] Nagai, K. et al. Data resource profile: JMDC claims databases sourced from medical institutions. *J. Gen. Fam. Med.* **21**, 211–218. 10.1002/jgf2.367 (2020). PMC7689231 33304714

[30] Tarabichi, Y. et al. The cosmos collaborative: a vendor-facilitated electronic health record data aggregation platform. *ACI Open* **5**, e36–e46. 10.1055/s-0041-1731004 (2021). PMC8775787 35071993

[31] Hsu, J. C., Yang, Y. Y., Chuang, S. L., Lee, J. K. & Lin, L. Y. Prediabetes increases the risk of major limb and cardiovascular events. *Cardiovasc. Diabetol.* **22**, 348. 10.1186/s12933-023-02085-y (2023). PMC10731780 38115080

[32] Wahab, A. et al. Phenotypic characterization of people at risk of atrial fibrillation: protocol for the FIND-AF longitudinal cohort study. *Eur. J. Prev. Cardiol.* **31**, 2099–2103. 10.1093/eurjpc/zwae303 (2024). PMC11663481 39319414

[33] Helbitz, A. et al. Identification, characterisation and outcomes of pre-atrial fibrillation in heart failure with reduced ejection fraction. *ESC Heart Fail.* 10.1002/ehf2.15347 (2025). PMC12450782 40528830

