# Supplementary material for: Scalable risk stratification of undiagnosed heart failure using routine health data and its association with imaging phenotypes and outcomes
Source: Sci Rep. 2026 Jul 11;16:31936. doi: 10.1038/s41598-026-60673-w (PMC13658026; doi:10.1038/s41598-026-60673-w)
Supplement: Supplementary file 1 [file 41598_2026_60673_MOESM1_ESM.docx]

**Supplementary Appendix**

Scalable risk stratification of undiagnosed heart failure using routine health data and its association with imaging phenotypes and outcomes

Yoko M. Nakao*, Ramesh Nadarajah*, Farag Shuweihdi, Christopher J. Hayward, Michihiko Goto, Jung-Chi Hsu, Mohammad Haris, Ben Hurdus, Osama Tariq, Temar Habtezghi, Anna Helbitz, Ali Wahab, Lan Mu, Kazuhiro Nakao, Peter Swoboda, Amitava Banerjee, Mark C. Petrie, Clare J Taylor, Koji Kawakami, Jianhua Wu, Chris Gale

Table of Contents

[Supplementary Methods	4](#_Toc229251326)

[Data sources and study populations	4](#_Toc229251327)

[Outcomes	6](#_Toc229251328)

[Sample size	7](#_Toc229251329)

[Patient and Public Involvement	8](#_Toc229251330)

[Supplementary Results	9](#_Toc229251331)

[Supplementary Table 1: Prediction performance of FIND-HF model, ARIC HF and PCP-HF in external validation cohorts stratified by sex	9](#_Toc229251332)

[Supplementary Table 2: Prediction performance of FIND-HF model for heart failure hospitalisation in NTUH	10](#_Toc229251333)

[Supplementary Table 3. Prediction performance of FIND-HF model for heart failure hospitalisation in NTUH stratified by sex	11](#_Toc229251334)

[Supplementary Table 4. Sensitivity, specificity, positive predictive value and negative predictive value at different thresholds for FIND-HF model	12](#_Toc229251335)

[Supplementary Table 5: Baseline characteristics of the participants in MATCH registry by the FIND-HF model score	13](#_Toc229251336)

[Supplementary Table 6. Summary statistics for outcomes stratified by higher versus lower FIND-HF risk	14](#_Toc229251337)

[Supplementary Table 7. Crude hazard ratios for outcomes between higher versus lower FIND-HF risk	15](#_Toc229251338)

[Supplementary Table 8. Summary statistics for outcomes stratified by higher versus lower FIND-HF risk – sensitivity analysis at 5 years	16](#_Toc229251339)

[Supplementary Table 9. Crude hazard ratios for outcomes between high versus low FIND-HF risk – sensitivity analysis at 5 years	17](#_Toc229251340)

[Supplementary Table 10. Cardiac MRI characteristics of the participants in MATCH registry by FIND-HF score	18](#_Toc229251341)

[Supplementary Table 11 Univariate linear regression showing the effect of higher FIND-HF score on raw cardiac MRI variables	19](#_Toc229251342)

[Supplementary Figure 1: Variable importance in logistic regression and linear discriminant analysis models	20](#_Toc229251343)

[Supplementary Figure 2. Kaplan-Meier plots of cardiovascular death and heart failure hospitalization by FIND-HF risk in the MATCH registry	21](#_Toc229251344)

[Supplementary Figure 3. Kaplan-Meier plots of the primary and secondary outcomes by FIND-HF risk score in the MATCH registry – sensitivity analysis at 5 years	22](#_Toc229251345)

[Supplementary Figure 4. Distribution of FIND-HF score by raw cardiac MRI parameters	23](#_Toc229251346)

[TRIPOD-AI Checklist	24](#_Toc229251347)

[References	26](#_Toc229251348)

# Supplementary Methods

## Data sources and study populations

***CPRD Aurum***

Clinical Practice Research Datalink (CPRD) Aurum is a primary care database launched in 2017 that comprises anonymised medical records and prescribing data contributed by general practices using EMIS Web software. It contains data for approximately 26.9 million patients and draws on data collected from practices in England only.^1^ Patient-level data is linked to Hospital Episode Statistics (HES) Admitted Patient Care (APC) and Office for National Statistics (ONS) Death Registration to provide a comprehensive coverage of diagnoses and outcomes. Scientific approval for this study was given by the CPRD Independent Scientific Advisory Committee (ISAC, ref no. 21_000324).

Participants were men and women registered at practices, ≥40 years of age at entry with no history of HF from and had at least 1 year of follow-up, between Jan 1, 1998, and Feb 28, 2022. Patients were eligible for inclusion if their record was labelled as acceptable by CPRD quality control,^2^ approved for CPRD and Hospital Episodes Statistics linkage, if they were registered with their general practice for at least 12 months, and if they had no prior history of heart failure (HF). Individuals were censored to a diagnosis of HF, withdrawal from CPRD, or 2 years, whichever came first.

***CPRD-GOLD***

CPRD-GOLD is a primary care database, established in 1987, that comprises anonymised medical records and prescribing data contributed by general practices using Vision software.^2^ It contains data for approximately 17.5 million patients, with 30% of contributing practices in England. The included patients are broadly representative of the UK general population regarding age, sex and ethnicity. Scientific approval for this study was given by the CPRD ISAC (ref no. 21_000324).

We included all men and women registered who were ≥40 years of age with no history of HF from and had at least 1 year of follow-up.

***JMDC***

The JMDC database is an epidemiological claims database that has compiled inpatient, outpatient, pharmacy, and dental claims, along with health check-up data, from multiple employer-based health insurance associations since 2005.^3^ An anonymized personal identifier enables the longitudinal linkage of claims across hospitals, clinics, and pharmacies, except in cases where individuals withdraw from or transition to a different insurer. The JMDC Claims Database consists predominantly of data from employee-based health insurance societies and their dependents, which tends to exclude retirees and may over-represent relatively health-conscious individuals. Consequently, the population might be healthier and younger than the general Japanese population, potentially limiting generalizability.^4^ This study was approved by Institutional Review Board at Kyoto University.(No. R4368).

We included all men and women registered who were ≥40 years of age with no history of HF from and had at least 1 year of follow-up.

***Epic Cosmos***

Epic Cosmos is a cloud-based data platform developed by Epic Systems to support the generation of real-world evidence and population health research. It aggregates de-identified electronic health record (EHR) data from a network of health systems that use Epic’s software. As of 2025, Cosmos includes data on more than 300 million patients who received care at over 1,700 hospitals and 40,000 clinics across the United States, Canada, Lebanon, and Saudi Arabia, making it one of the largest collections of longitudinal clinical data globally. All patient data in Cosmos is de-identified in accordance with Health Insurance Portability and Accountability Act (HIPAA) regulations and standardised using common clinical terminologies. Access to Cosmos is limited to participating institutions that contribute data, and use is governed by strict data use and compliance policies. Researchers can query the data using Epic’s built-in tools or relational database system exclusively in approved environments.

We included all men and women who were ≥40 years of age with no history of HF, had primary care visits and were followed for at least 1 year during the study period in the United States. The predictors were assessed at the time of the first primary care clinic visit for each patient, and the onset of HF was defined as the use of ICD-10 code for HF within 1 year of the first primary care clinic visit.

***NTUH***

We used EHRs from the National Taiwan University Hospital Integrated Medical Database (NTUH-iMD).^5^ Prior research has established the consistent quality of this medical database.^6^ The NTUH-iMD is a comprehensive data collection system that aggregates information from Taipei Main Hospital and its affiliated branches in at least four counties across Taiwan. These digitized EHRs have been accessible through online platforms since 2006 and contain a wide range of patient-related information, including demographics, diagnoses, physician prescriptions, laboratory results, interventions, medications, and examinations. This study was approved by Institutional Review Board (202410100RINA).

We included all men and women who were ≥40 years of age at entry with no history of HF and had at least 1 year of follow-up.

***FIND-AF cohort***

The FIND-AF longitudinal cohort included community-based patients with a primary care EHR were as at risk of atrial fibrillation by the FIND-AF algorithm from their primary care EHR data, aged 30 years and above and eligible for oral anticoagulation (men with a CHA_2_DS_2_-VASc score ≥2 or women with a CHA_2_DS_2_-VASc score ≥3), and recruited between October 2023 and November 2024 to receive home-based intermittent electrocardiogram monitoring in the FIND-AF study.^7,8^ Participants with higher FIND-AF score in the study but without diagnosed atrial fibrillation during electrocardiogram monitoring were then included in the FIND-AF longitudinal cohort then underwent cross-sectional phenotypic characterisation including physical examination, symptoms assessment, serum blood biomarkers and echocardiography.^9^ Ethical approval was given by the North West – Greater Manchester South Research Ethics Committee (reference 23/NW/0180).

We included all participants without a background history of HF who underwent symptom assessment, natriuretic peptide testing and transthoracic echocardiography.

***MATCH 1 and 2 registry***

Patients who were clinically diagnosed with heart failure at cardiology clinics and referred for cardiac MRI assessment were recruited into the MATCH (MyocArdial Tissue CHaracteristics in patients with HF according to glycaemic status) 1 and 2 registries between 2018 and 2024. Patients were classified to have heart failure with reduced ejection fraction (HFrEF) if found to have a baseline LVEF ≤40% on echocardiography at the time of diagnosis. Patients were excluded if they had a baseline LVEF >40%, a known history of coronary artery disease (>70% on invasive coronary angiography), myocardial infarction, coronary revascularisation or anginal symptoms. Other exclusion criteria included hypertrophic cardiomyopathy, amyloidosis, congenital heart disease and advanced renal failure. Cardia MRI images were acquired in a supine position using a 3T system (Siemens Magnetom Prisma, Erlangen, Germany). The study protocol included cine imaging, stress-perfusion imaging and motion-corrected bright-blood late gadolinium enhancement (LGE). For stress imaging, adenosine was infused for at least 3 min, at a rate of 140 g/kg/min and if there was insufficient haemodynamic response (increase in heart rate <10 beats/min) or lack of symptomatic response, infused at an increased rate of up to 210 g/kg/min. LGE images were acquired as a short-axis stack, and in four-chamber, three-chamber and two-chamber views. When it was unclear if any enhancement on bright-blood LGE represented ischaemic changes, a dark-blood LGE stack was acquired. Image analysis was carried out on the cvi42 software (Circle Cardiovascular Imaging, Calgary Canada). This research was approved by the National Research Ethics Committee (17/YH/0300 and 20/NW/0326).

We included all participants aged ≥40 years of age, all of whom had received a HF diagnosis.

## Outcomes

The primary outcome was a first record of HF in primary care or hospital admission records.

***Incident heart failure***

In CPRD Aurum and GOLD, we defined incident HF as the first record of HF in primary care (using Read codes) or hospital admission records (using the International Classification of Diseases, tenth revision [ICD-10]) from any diagnostic position using a comprehensive set of diagnostic codes.^10,11,12^ In JMDC, Epic Cosmos and NTUH we used the same ICD-10 codes of CPRD.

***FIND-AF cohort***

In the FIND-AF cohort diagnoses of asymptomatic left ventricular systolic dysfunction (LVSD), HF with reduced ejection fraction, HF with mid-range ejection fraction and HF with preserved ejection fraction, in accordance with European society of cardiology guidelines using symptom assessment, natriuretic peptide testing, and transthoracic echocardiography.^13^

***MATCH registry***

The primary outcome was time to a composite of major adverse cardiovascular events (HF hospitalisation, all-cause mortality, stroke and myocardial infarction) as derived from participant-level medical records. Secondary outcomes included individual components of the primary outcome, cardiovascular death, and association of cardiac MRI variables with FIND-HF score.

## Sample size

We calculated sample size with R package pmsampsize and pvalsampsize, from Riley et al.^14,15^. For 20 parameters to be included in the prediction model and the Cox-Snell generalised R^2^ is assumed to be 0.05. A total of 3699 patients will be required to meet Riley’s criterion (1) with global shrinkage factor of 0.95; this sample size also ensures a small absolute difference (Δ<0.05) in the apparent and adjusted Nagelkerke R2 (Riley’s criterion (2)) and ensures precise estimate of overall risk with a margin of error <0.001 (Riley’s criterion (3)). In the derivation cohort there were 3 520 186 participants with 37 094 new HF cases and in the validation cohorts there were 7 391 544 participants with 54 029 new HF cases new AF cases. Therefore, the number of patients provided sufficient statistical power to develop and validate a multivariable prediction model with the predefined precision and accuracy.

## Patient and Public Involvement

No patient or public contributors were involved in the design, conduct, reporting, or dissemination plans for this research.

# Supplementary Results

## Supplementary Table 1: Prediction performance of FIND-HF model, ARIC HF and PCP-HF in external validation cohorts stratified by sex

|  | **FIND-HF** | **PCP-HF** | **ARIC-HF** |
| --- | --- | --- | --- |
|  | **AUROC (95% CI)** | **AUROC (95% CI)** | **AUROC (95% CI)** |
| **CPRD-GOLD, UK** |  |  |  |
| Overall | 0·722 (0·715 – 0·728) | 0·605 (0·509 – 0·700) | 0·612 (0·545 – 0·679) |
| Men | 0·714 (0·705 – 0·724) | 0·632 (0·488 – 0·775) | 0·632 (0·513 – 0·752) |
| Women | 0·730 (0·721 – 0·739) | 0·583 (0·453 – 0·713) | 0·664 (0·579 – 0·749) |
| **JMDC, Japan** |  |  |  |
| Overall | 0·728 (0·726 – 0·730) | 0·702 (0·698 – 0·707) | – |
| Men | 0·744 (0·741 – 0·747) | 0·710 (0·705 – 0·715) | – |
| Women | 0·703 (0·699 – 0·707) | 0·670 (0·661 – 0·678) | – |
| **Epic Cosmos, US** |  |  |  |
| Overall | 0·784 (0·782 – 0·785) | – | – |
| Men | 0·772 (0·770 – 0·774) | – | – |
| Women | 0·790 (0·788 – 0·792) | – | – |

ARIC, Atherosclerosis Risk in Communities; AUROC, area under received operating characteristic; CI, confidence interval; CPRD, Clinical Practice Research Datalink; FIND-HF, Future Innovations in Novel Detection of Heart Failure; LDA, Linear Discriminant Analysis; PCP-HF, Pooled Cohort Equations to Prevent Heart Failure.

## Supplementary Table 2: Prediction performance of FIND-HF model for heart failure hospitalisation in NTUH

|  | **FIND-HF, logistic regression** |
| --- | --- |
| AUROC (95% CI) | 0·850 (0·836 – 0·864) |
| Brier score | 0·005 |
| Calibration slope | 1·349 |

AUROC, area under received operating characteristic; CI, confidence interval; CPRD, Clinical Practice Research Datalink; FIND-HF, Future Innovations in Novel Detection of Heart Failure; LDA, Linear Discriminant Analysis.

## Supplementary Table 3. Prediction performance of FIND-HF model for heart failure hospitalisation in NTUH stratified by sex

|  | **FIND-HF** |
| --- | --- |
|  | **AUROC (95% CI)** |
| Overall | 0·850 (0·836 – 0·864) |
| Men | 0·829 (0·810 – 0·848) |
| Women | 0·881 (0·863 – 0·899) |

AUROC, area under received operating characteristic; CI, confidence interval; FIND-HF, Future Innovations in Novel Detection of Heart Failure.

## Supplementary Table 4. Sensitivity, specificity, positive predictive value and negative predictive value at different thresholds for FIND-HF model

|  | **Threshold** | **Sensitivity** | **Specificity** | **PPV** | **NPV** | **F1 score** |
| --- | --- | --- | --- | --- | --- | --- |
| FIND-HF | 0·0097 | 1·000 | 0·205 | 0·148 | 1·000 | 0.258 |
|  | 0·0105 | 1·000 | 0·272 | 0·160 | 1·000 | 0.276 |
|  | 0·0125 | 0·968 | 0·272 | 0·182 | 0·980 | 0.306 |
|  | 0·0149 | 0·871 | 0·554 | 0·214 | 0·969 | 0.344 |

FIND-HF, Future Innovations in Novel Detection of Heart Failure; NPV, negative predictive value; PPV, positive predictive value.

## Supplementary Table 5: Baseline characteristics of the participants in MATCH registry by the FIND-HF model score

|  | **Total**  **(N=553)** | **High**  **FIND-HF risk (N=263)** | **Low**  **FIND-HF risk (N=290)** | **p value** |
| --- | --- | --- | --- | --- |
| Age, years | 64.40 ± 10.33 | 70.77 ± 7.781 | 58.62 ± 8.850 | <0.001 |
| Women (%) | 192 (34.7) | 83 (31.6) | 109 (37.6) | 0.416 |
| Body mass index, kg/m^2^ | 29.47 ± 11.59 | 29.09 ± 12.46 | 29.81 ± 10.77 | 0.691 |
| Hypertension (%) | 253 (45.8) | 132 (50.2) | 121 (41.7) | 0.266 |
| Diabetes mellitus (%) | 112 (20.3) | 58 (22.1) | 54 (18.6) | 0.608 |
| Vascular disease (%) | 130 (23.5) | 100 (38.0) | 30 (10.3) | <0.001 |
| Chronic obstructive pulmonary disease (%) | 49 (8.9) | 40 (15.2) | 9 (3.1) | 0.009 |
| Valvular heart disease (%) | 208 (37.6) | 154 (58.6) | 54 (18.6) | <0.001 |
| Stroke (%) | 62 (11.2) | 36 (13.7) | 26 (9.0) | 0.367 |
| Chronic kidney disease (%) | 26 (4.7) | 16 (6.1) | 10 (3.4) | 0.502 |
| Rheumatoid arthritis (%) | 6 (1.1) | 3 (1.1) | 3 (1.0) | 1.000 |

FIND-HF, Future Innovations in Novel Detection of Heart Failure.

## Supplementary Table 6. Summary statistics for outcomes stratified by higher versus lower FIND-HF risk

|  | **Total (N=553)** | **High FIND-HF risk (N=263)** | **Low FIND-HF risk (N=290)** | **p value** |
| --- | --- | --- | --- | --- |
| **Heart failure hospitalisation or CV Death**  Over median 2.58 years (IQR: 1.42-5.17 years) follow up | 62 | 38 | 24 | 0.024 |
| **Heart failure hospitalisation** | 52 | 32 | 20 | 0.038 |
| **Cardiovascular death** | 21 | 11 | 10 | 0.661 |

FIND-HF, Future Innovations in Novel Detection of Heart Failure; MACE, major adverse cardiovascular events.

## Supplementary Table 7. Crude hazard ratios for outcomes between higher versus lower FIND-HF risk

|  | **Hazard Ratio (95% CI)** | **p value** |
| --- | --- | --- |
| **Heart failure hospitalisation or cardiovascular Death** | 1.78 (1.07, 2.98) | 0.026 |
| **Heart failure hospitalisation** | 1.79 (1.03, 3.13) | 0.041 |
| **Cardiovascular death** | 1.21 (0.51, 2.85) | 0.661 |

CI, confidence interval; FIND-HF, Future Innovations in Novel Detection of Heart Failure;

## Supplementary Table 8. Summary statistics for outcomes stratified by higher versus lower FIND-HF risk – sensitivity analysis at 5 years

|  | **Total (N=553)** | **High FIND-HF risk (N=263)** | **Low FIND-HF risk (N=290)** | **p value** |
| --- | --- | --- | --- | --- |
| **HF Hospitalisation/CV Death** | 60 | 37 | 23 | 0.022 |
| **Heart failure hospitalisation** | 50 | 31 | 19 | 0.035 |
| **CV death** | 21 | 11 | 10 | 0.661 |

## Supplementary Table 9. Crude hazard ratios for outcomes between high versus low FIND-HF risk – sensitivity analysis at 5 years

|  | **Hazard Ratio (95% CI)** | **p value** |
| --- | --- | --- |
| **HF Hospitalisation/CV Death** | 1.82 (1.08, 3.06) | 0.024 |
| **HF hospitalisation** | 1.83 (1.04, 3.25) | 0.037 |
| **CV Death** | 1.21 (0.51, 2.85) | 0.661 |

## Supplementary Table 10. Cardiac MRI characteristics of the participants in MATCH registry by FIND-HF score

|  | **Total**  **(N=553)** | **Higher**  **FIND-HF risk (N=263)** | **Lower**  **FIND-HF risk (N=290)** | **p value** |
| --- | --- | --- | --- | --- |
| **Clinical cardiac magnetic resonance** | | | | |
| Left atrial volume (ml) | 90.09 ± 44.10 | 101.3 ± 48.79 | 79.99 ± 36.67 | 0.002 |
| Left atrial volume indexed to BSA (ml) | 45.69 ± 21.25 | 51.75 ± 23.12 | 40.24 ± 17.77 | <0.001 |
| Left ventricular ejection fraction (%) | 38.78 ± 12.90 | 36.84 ± 12.19 | 40.55 ± 13.29 | 0.038 |
| Left ventricular mass (g) | 133.7 ± 43.47 | 133.0 ± 43.08 | 134.4 ± 43.89 | 0.815 |
| Left ventricular mass indexed to BSA (g/m^2^) | 67.96 ± 20.05 | 68.72 ± 20.77 | 67.28 ± 19.38 | 0.626 |
| Pulmonary capillary wedge pressure (mmHg) | 14.91 ± 2.79 | 15.29 ± 2.91 | 14.58 ± 2.64 | 0.084 |
| Right ventricular ejection fraction (%) | 49.03 ± 12.04 | 46.63 ± 12.18 | 51.20 ± 11.51 | 0.009 |
| Left ventricular end-diastolic volume (ml) | 217.0 ± 76.60 | 215.5 ± 74.98 | 218.3 ± 78.15 | 0.795 |
| Left ventricular end-diastolic volume indexed to BSA (ml) | 110.8 ± 38.78 | 111.7 ± 38.36 | 109.9 ± 39.21 | 0.738 |
| Right ventricular end-diastolic volume indexed to BSA (ml) | 80.48 ± 24.30 | 81.35 ± 25.21 | 79.69 ± 23.45 | 0.643 |
| **Tissue characteristics** | | | | |
| Ischaemic fibrosis (%) | 116 (21.0) | 80 (30.4) | 36 (12.4) | 0.004 |
| Non-ischaemic fibrosis (%) | 172 (31.1) | 87 (33.1) | 85 (29.3) | 0.587 |
| Inducible ischaemia (%) | 43 (7.8) | 34 (12.9) | 9 (3.1) | 0.024 |
| Native T1 (ms) | 1332 ± 54.10 | 1338 ± 56.18 | 1327 ± 51.76 | 0.184 |
| Extracellular volume (%) | 27.71 ± 4.78 | 28.35 ± 5.15 | 27.14 ± 4.35 | 0.113 |

BSA, body surface area.

## Supplementary Table 11 Univariate linear regression showing the effect of higher FIND-HF score on raw cardiac MRI variables

|  | **β** **(95% CI)** | **p value** |
| --- | --- | --- |
| **Clinical cardiac magnetic resonance** | | |
| Left atrial volume (ml) | 21.33 (14.13, 28.54) | < 0.001 |
| Left atrial volume indexed to BSA (ml) | 11.51 (8.07, 14.95) | < 0.001 |
| Left ventricular ejection fraction (%) | -3.71 (-5.86, -1.56) | < 0.001 |
| Left ventricular mass (g) | -1.45 (-8.78, 5.88) | 0.698 |
| Left ventricular mass indexed to BSA (g/m^2^) | 1.44 (-1.94, 4.82) | 0.403 |
| Pulmonary capillary wedge pressure (mmHG) | 0.71 (0.25, 1.18) | 0.003 |
| Right ventricular ejection fraction (%) | -4.57 (-6.57, -2.57) | < 0.001 |
| **Tissue characteristics** | | |
| Native T1 (ms) | 10.63 (1.51, 19.74) | 0.022 |
| Extracellular volume (%) | 1.21 (0.37, 2.06) | 0.005 |

BSA, body surface area; CI, confidence interval; FIND-HF, Future Innovations in Novel Detection of Heart Failure.

## Supplementary Figure 1: Variable importance in logistic regression and linear discriminant analysis models

LDA = Linear Discriminant Analysis.

## Supplementary Figure 2. Kaplan-Meier plots of cardiovascular death and heart failure hospitalization by FIND-HF risk in the MATCH registry

HF, heart failure; CV, cardiovascular; LR, logistic regression; MATCH, MyocArdial Tissue CHaracteristics in patients with HF according to glycaemic status.

## Supplementary Figure 3. Kaplan-Meier plots of the primary and secondary outcomes by FIND-HF risk score in the MATCH registry – sensitivity analysis at 5 years

CV, cardiovascular; HF, heart failure; LR, logistic regression

## Supplementary Figure 4. Distribution of FIND-HF score by raw cardiac MRI parameters

ECV, extracellular volume fraction; FIND-HF, Future Innovations in Novel Detection of Heart Failure; LA, left atrium; LR, logistic regression; LV, left ventricular; LVEDV, left ventricular end-diastolic volume; LVEF, left ventricular ejection fraction; PCWP, pulmonary capillary wedge pressure; RV, right ventricular; RVEDV, right ventricular end-diastolic volume

## TRIPOD-AI Checklist

| **Section/Topic** | **Item** | **Development/Evaluation** | **Checklist Item** | **Reported** |
| --- | --- | --- | --- | --- |
| TITLE | 1 | D;E | Identify the study as developing or evaluating the performance of a multivariable prediction model, the target population, and the outcome to be predicted | Title |
| ABSTRACT | 2 | D;E | See TRIPOD+AI for Abstracts checklist | Abstract |
| INTRODUCTION | 3a | D;E | Explain the healthcare context and rationale for developing or evaluating the prediction model | Introduction |
| INTRODUCTION | 3b | D;E | Describe the target population and intended purpose in the care pathway | Introduction |
| INTRODUCTION | 3c | D;E | Describe any known health inequalities between sociodemographic groups | Supplementary methods |
| INTRODUCTION | 4 | D;E | Specify study objectives, including development or validation (or both) | Introduction |
| METHODS - Data | 5a | D;E | Describe data sources, rationale, and representativeness | Methods - Data source and study populations |
| METHODS - Data | 5b | D;E | Specify dates of data collection | Methods - Data source and study populations |
| METHODS - Participants | 6a | D;E | Specify study setting and number/location of centres | Methods – study design |
| METHODS - Participants | 6b | D;E | Describe eligibility criteria | Methods - Data source and study populations |
| METHODS - Participants | 6c | D;E | Give details of treatments received and how handled | NA |
| METHODS - Data preparation | 7 | D;E | Describe pre-processing and quality checking | Supplementary methods |
| METHODS - Outcome | 8a | D;E | Define the outcome, time horizon, assessment method | Method – outcome, Supplementary methods - outcome |
| METHODS - Outcome | 8b | D;E | Describe outcome assessor qualifications and demographics | Method – outcome, Supplementary methods - outcome |
| METHODS - Outcome | 8c | D;E | Report actions to blind outcome assessment | NA |
| METHODS - Predictors | 9a | D | Describe choice of initial predictors | Methods - Predictor variables |
| METHODS - Predictors | 9b | D;E | Define all predictors, assessment method and blinding | Methods - Predictor variables |
| METHODS - Predictors | 9c | D;E | Describe predictor assessor qualifications and demographics | NA |
| METHODS - Sample size | 10 | D;E | Explain and justify study size | Supplementary method |
| METHODS - Missing data | 11 | D;E | Describe handling of missing data | No missing data for FIND-HF |
| METHODS - Analytical methods | 12a | D | Describe how data were used in analysis, including data partitioning | Methods - Data source and study populations |
| METHODS - Analytical methods | 12b | D | Describe handling of predictors in analysis | Methods -  Predictor variables |
| METHODS - Analytical methods | 12c | D | Specify model type, rationale, building steps, internal validation | Methods - Model development and internal validation |
| METHODS - Analytical methods | 12d | D;E | Describe handling of heterogeneity in parameters and performance | Supplementary Methods- Data source and study populations |
| METHODS - Analytical methods | 12e | D;E | Specify all measures and plots to evaluate performance | Methods - Predictive modelling and model performance |
| METHODS - Analytical methods | 12f | E | Describe any model updating | NA |
| METHODS - Analytical methods | 12g | E | Describe how predictions were calculated | Methods - International external validation |
| METHODS - Class imbalance | 13 | D;E | Describe use and rationale for class imbalance methods | NA |
| METHODS - Fairness | 14 | D;E | Describe fairness methods and rationale | Methods - Predictive modelling and model performance |
| METHODS - Model output | 15 | D | Specify model output and classification threshold rationale | Methods - Predictive modelling and model performance |
| METHODS - Training vs Evaluation | 16 | D;E | Identify differences between development and evaluation datasets | Table 1 |
| METHODS - Ethical approval | 17 | D;E | Provide IRB/ethics approval and informed consent information | Supplemental Methods - Data sources and study populations |
| OPEN SCIENCE - Funding | 18a | D;E | Give source and role of funding | Methods - Role of the funding source |
| OPEN SCIENCE - Conflicts of interest | 18b | D;E | Declare conflicts of interest | Declaration of Interests |
| OPEN SCIENCE - Protocol | 18c | D;E | Indicate protocol accessibility | Published:  Nakao YM, et al. BMJ Open. 2024;14(1):e073455. |
| OPEN SCIENCE - Registration | 18d | D;E | Provide registration details | NA |
| OPEN SCIENCE - Data sharing | 18e | D;E | Provide data availability details | Data sharing |
| OPEN SCIENCE - Code sharing | 18f | D;E | Provide code availability details | Data sharing |
| PATIENT & PUBLIC INVOLVEMENT | 19 | D;E | Describe patient/public involvement or state none | Supplementary methods - Patient and Public Involvement |
| RESULTS - Participants | 20a | D;E | Describe participant flow, outcome status, follow-up | Figure 1 |
| RESULTS - Participants | 20b | D;E | Report characteristics by source, setting, or group | Table 1 |
| RESULTS - Participants | 20c | E | Compare predictor distributions in development vs evaluation | Table 2 |
| RESULTS - Model development | 21 | D;E | Specify number of participants/events in each analysis | Table 1 |
| RESULTS - Model specification | 22 | D | Provide prediction model details for implementation | Results - International external validation |
| RESULTS - Model performance | 23a | D;E | Report performance with confidence intervals | Table 2-3, Figure 2 |
| RESULTS - Model performance | 23b | D;E | Report heterogeneity in model performance | Table 2-3, Figure 2 |
| RESULTS - Model updating | 24 | E | Report updated model and its performance | NA |
| DISCUSSION - Interpretation | 25 | D;E | Interpret results including fairness issues | Discussion |
| DISCUSSION - Limitations | 26 | D;E | Discuss limitations, biases, uncertainty, generalizability | Discussion |
| DISCUSSION - Usability | 27a | D | Describe handling of poor or missing input data | No missing data for FIND-HF model |
| DISCUSSION - Usability | 27b | D | Specify user interaction and expertise required | Discussion |
| DISCUSSION - Usability | 27c | D;E | Discuss next steps for applicability/generalizability | Discussion |

# References

1.	Wolf A, Dedman D, Campbell J, et al. Data resource profile: Clinical Practice Research Datalink (CPRD) Aurum. *International Journal of Epidemiology* 2019; **48**(6): 1740-g.

2.	Herrett E, Gallagher AM, Bhaskaran K, et al. Data resource profile: clinical practice research datalink (CPRD). *International journal of epidemiology* 2015; **44**(3): 827-36.

3.	Nagai K, Tanaka T, Kodaira N, Kimura S, Takahashi Y, Nakayama T. Data resource profile: JMDC claims databases sourced from Medical Institutions. *J Gen Fam Med* 2020; **21**(6): 211-8.

4.	Laurent T, Simeone J, Kuwatsuru R, et al. Context and Considerations for Use of Two Japanese Real-World Databases in Japan: Medical Data Vision and Japanese Medical Data Center. *Drugs Real World Outcomes* 2022; **9**(2): 175-87.

5.	Hsu J-C, Yang Y-Y, Chuang S-L, Lee J-K, Lin L-Y. Prediabetes increases the risk of major limb and cardiovascular events. *Cardiovascular Diabetology* 2023; **22**(1): 348.

6.	Lee Y-C, Chao Y-T, Lin P-J, et al. Quality assurance of integrative big data for medical research within a multihospital system. *Journal of the Formosan Medical Association* 2022; **121**(9): 1728-38.

7.	Nadarajah R, Wahab A, Reynolds C, et al. Future Innovations in Novel Detection for Atrial Fibrillation (FIND-AF): pilot study of an electronic health record machine learning algorithm-guided intervention to identify undiagnosed atrial fibrillation. *Open Heart* 2023; **10**(2): e002447.

8.	Nadarajah R, Wu J, Hogg D, et al. Prediction of short-term atrial fibrillation risk using primary care electronic health records. *Heart* 2023; **109**(14): 1072-9.

9.	Wahab A, Nadarajah R, Reynolds C, et al. Phenotypic characterization of people at risk of atrial fibrillation: protocol for the FIND-AF longitudinal cohort study. *European Journal of Preventive Cardiology* 2024; **31**(18): 2099-103.

10.	Conrad N, Judge A, Tran J, et al. Temporal trends and patterns in heart failure incidence: a population-based study of 4 million individuals. *Lancet* 2018; **391**(10120): 572-80.

11.	Conrad N, Judge A, Canoy D, et al. Temporal Trends and Patterns in Mortality After Incident Heart Failure: A Longitudinal Analysis of 86 000 Individuals. *JAMA Cardiol* 2019; **4**(11): 1102-11.

12.	Nakao YM, Nakao K, Nadarajah R, et al. Prognosis, characteristics, and provision of care for patients with the unspecified heart failure electronic health record phenotype: a population-based linked cohort study of 95262 individuals. *EClinicalMedicine* 2023; **63**: 102164.

13.	Members: ATF, McDonagh TA, Metra M, et al. 2021 ESC Guidelines for the diagnosis and treatment of acute and chronic heart failure: Developed by the Task Force for the diagnosis and treatment of acute and chronic heart failure of the European Society of Cardiology (ESC). With the special contribution of the Heart Failure Association (HFA) of the ESC. *European journal of heart failure* 2022; **24**(1): 4-131.

14.	Riley RD, Snell KI, Ensor J, et al. Minimum sample size for developing a multivariable prediction model: PART II‐binary and time‐to‐event outcomes. *Statistics in medicine* 2019; **38**(7): 1276-96.

15.	Riley RD, Snell KI, Archer L, et al. Evaluation of clinical prediction models (part 3): calculating the sample size required for an external validation study. *bmj* 2024; **384**.
